# Dynamics of a type 2 secretion system pseudopilus unraveled by complementary approaches

**DOI:** 10.1007/s10858-019-00246-4

**Published:** 2019-05-23

**Authors:** Benjamin Bardiaux, Florence Cordier, Sébastien Brier, Aracelys López-Castilla, Nadia Izadi-Pruneyre, Michael Nilges

**Affiliations:** 1 Structural Bioinformatics Unit, Department of Structural Biology and Chemistry, C3BI, Institut Pasteur; CNRS UMR3528; CNRS USR3756, Paris, France; 2 Biological NMR Technological Platform, Center for Technological Resources and Research, Department of Structural Biology and Chemistry, Institut Pasteur; CNRS UMR3528, Paris, France

**Keywords:** Pili, Dynamics, NMR, HDX-MS, Cryo-EM, T2SS

## Abstract

Secretion pili, bacterial fibers responsible for transporting proteins to the extracellular milieu in some secretion systems, are very strong structures but at the same time highly flexible. Their flexibility and helical symmetry make structure determination at atomic resolution a challenging task. We have previously used an integrative structural biology approach including liquid-state NMR, cryo-electron microscopy (cryo-EM), and modeling to determine the pseudo-atomic resolution structure of the type 2 secretion system pseudopilus in a mutant form, where we employed NMR to determine the high resolution structure of the pilin (the monomer building block of the pilus). In this work, we determine the pseudo-atomic structure of the wild type pilus, and compare the dynamics of wild type and mutant pili by normal mode analysis. We present a detailed NMR analysis of the dynamics of the pilin in isolation, and compare dynamics and solvent accessibility of isolated and assembled pilins by Hydrogen/Deuterium eXchange Mass Spectrometry (HDX-MS). These complementary approaches provide a comprehensive view of internal and overall dynamics of pili, crucial for their function.

**Electronic supplementary material:**

The online version of this article (10.1007/s10858-019-00246-4) contains supplementary material, which is available to authorized users.

## Introduction

Secretion systems are essential for bacteria to transport substrates across membranes. The type II secretion system (T2SS) enables many Gram-negative bacteria to secrete specific folded proteins from the periplasmic space to the extracellular milieu. The transport is accomplished by the assembly of a thin filament, the pseudopilus, formed by numerous copies of small proteins called pilins. This complex system is related to archaeal flagella, and type IV pili (T4P), flexible filaments promoting bacterial motility and adherence to surfaces and various host cells (Giltner et al. 2012). In the T2SS, in contrast to the flagella and T4P, the filament does not extend into the extracellular milieu but disassembles when it reaches the outer membrane, and is hence called pseudopilus. However, in the T2SS from *Klebsiella oxytoca*, it can extend beyond the bacterial surface and form a pilus when the major pilin PulG is overproduced. Similar to the flagella and T4P, these pili are highly flexible and at the same time resistant to force (Thomassin et al. 2017).

A considerable amount of work has gone into the study of the structures of T2SS pili and T4Ps in order to understand the transport mechanism and other functions in detail. Pili are not amenable to X-ray crystallography since their helical symmetry is incommensurate with crystal symmetry. In addition, even with latest generation electron microscopes (EM) and cameras, it has so far been impossible to obtain an atomic resolution structure of a T2SS pseudopilus or T4 pilus (Wang et al. 2017; Kolappan et al. 2016; López-Castilla et al. 2017; Bardiaux et al. 2019). In the known full-length X-ray crystal structures of the major T4 pilin (Parge et al. 1995; Craig et al. 2003; Hartung et al. 2011), the overall architecture of the pilin comprises a long, essentially uninterrupted N-terminal α helix of around 50 residues (α1) and a globular head. Around 20 residues at the C-terminal end of α1 are folded onto the globular head, whereas the first 30 residues, including a strongly hydrophobic, highly conserved N-terminal segment of around 20 residues (α1-N), protrude. Before assembling into a pilus, pilins are anchored in the inner membrane by α1-N. By sequence similarity, all pilins from T2SS and T4P are expected to share this general architecture.

The hydrophobicity of α1-N makes structural studies of isolated full-length pilins challenging. Hence, they are usually truncated and α1-N is not included. There are only very few examples of structures of complete pilins available (Parge et al. 1995; Craig et al. 2003; Hartung et al. 2011). In these structures, α1 is S-shaped and has only two very short interruptions, at residues 22 and 42, where the helix is slightly bent. Secondary structure predictions, however, suggest a less structured region of very low helix propensity around residue 20, at the end of α1-N.

The first structure of a T2SS pilin, the major pilin PulG not including α1-N, did not contain calcium (Köhler et al. 2004), which induced misfolding of the C-terminal region of the pilin, close to the actual calcium binding site. This led to a non-physiological dimer with a domain-swapped C-terminal region. Comparative modeling of α1-N and remodeling of the C-terminal region made it possible to build a first qualitative model of the PulG pilus, chosen to be left-handed (Köhler et al. 2004). Structures of homologous pilins containing calcium (Korotkov et al. 2009) allowed us to model a pilin with a correctly formed calcium binding site (Campos et al. 2010).

Based on this model, a first detailed, experimentally validated structure of the PulG pilus was generated from low resolution EM data by a flexible docking approach (Campos et al. 2010, 2011). EM measurements (Köhler et al. 2004) provided constraints on the helical arrangement of the pilins, but not the handedness. Further structural restraints were obtained from mutation experiments (charge inversions, double-cysteine substitutions in the transmembrane segment). These restraints improved convergence and helped to determine the handedness (Campos et al. 2011). In the pilus, PulG is organized in a 1-start right-handed helix, consistent with the organization of pilins in the gonococcal T4P (Craig et al. 2006). To take into account the symmetry ambiguity of inter-residue contacts derived from mutation experiments, we developed a novel method (Campos et al. 2011) which was then further extended and combined with the Ambiguous Restraints for Iterative Assignment (ARIA) approach (Rieping et al. 2007) to incorporate NMR data from solid and liquid state NMR experiments (Bardiaux et al. 2012; He et al. 2016).

In contrast to the gonococcal T4P obtained by rigidly docking the structure of the pilin into the EM density, our models showed substantial structural heterogeneity, in particular in side-chain positions. These dynamic aspects were further explored by explicitly incorporating the heterogeneity of helical parameters observed in EM into the modeling approach (Nivaskumar et al. 2014). This led to a detailed description of the importance of different inter-protomer interactions in the assembly of the pilus.

Recently, we determined a high resolution liquid-state NMR structure of the N-terminally truncated pilin in the presence of calcium, and investigated the critical role of calcium for folding, assembly, dynamics, and stability of individual pilins and of the assembled pilus (López-Castilla et al. 2017). Initial EM reconstructions were limited to about 7.5 Å in resolution due to the pronounced flexibility of the pili. The NMR structure and dynamics suggested the possibility to stabilize the pilin by replacing a hydrophobic interaction in the C-terminal region of the pilin by a disulfide bridge, which resulted in a more ordered, albeit less stable filament (named PulG_CC_), and improved the resolution of the cryo-EM reconstruction to 5 Å. This allowed us to determine a pseudo-atomic structure of the mutant pilus. This structure shows a discontinuity in α1 extending over several residues at the C-terminal end of α1-N, similar to what is observed in other recently determined structures of T4P (Wang et al. 2017; Kolappan et al. 2016) and corresponding to the predicted region of low helical propensity.

In this paper we investigate the intricate interplay of the structure and dynamics of isolated pilins and assembled pili. We present the pseudo-atomic structure of the wild type pilus, and compare the dynamics of the wild type and mutant pili by normal mode analysis. We present a detailed NMR analysis of the dynamics of the monomeric pilin. Normal mode analysis allows us to relate local pilin structure to the overall flexibility of the pilus, and Hydrogen/Deuterium eXchange Mass Spectrometry (HDX-MS), to compare dynamics and solvent accessibility of monomeric and assembled pilins.

## Materials and methods

### Refinement of wild-type pilus model in cryo-EM density

To obtain an initial model of the wild-type PulG pilus (PulG_WT_), cysteines at positions 106 and 129 in the PulG_CC_ pilus structure (PDB 5WDA) were first mutated in silico to their respective amino-acids in the PulG wild-type sequence (mutations C106H and C129W) with Modeller (Webb and Sali 2016). A subset of the PulG_WT_ pilus models, i.e. ten consecutive PulG subunits along the 1-start helix, was then docked into the PulG_WT_ cryo-EM reconstruction (López-Castilla et al. 2017) with Situs (Wriggers 2012). The resolution of the PulG_WT_ cryo-EM reconstruction was estimated at ~ 7.5 Å from model:map FSC analysis with the PulG_CC_ structure (López-Castilla et al. 2017). Ten additional perturbed models of the initial PulG_WT_ pilus model were generated as regularly spaced snapshots from a short cartesian molecular dynamics (MD) simulation performed with Phenix (Adams et al. 2010). The final root mean square deviation (RMSD) on Cα atoms of the MD simulation with the initial PulG_WT_ pilus model was 1.74 Å. Finally, the 11 PulG_WT_ pilus models obtained (one initial + 10 perturbed) were refined in the PulG_WT_ cryo-EM reconstruction with Phenix. The protocol for real-space refinement included global minimization and morphing with NCS and Ramachandran restraints (Afonine et al. 2018). For comparison purpose, the same protocol (MD + real-space refinement) was applied to the PulG_CC_ structure (PDB 5WDA), using the PulG_CC_ cryo-EM reconstruction at 5 Å resolution (EMD-8812, cropped along Z-axis, i.e., the pilus axis, to have the same dimension as the PulG_WT_ density map). Model:map cross-correlation coefficients were computed with TEMPy (Farabella et al. 2015). Model comparison was performed by superimposition of the pilus central subunits and computation of average pairwise RMSD on Cα atoms. Regions with significant RMSD differences (ΔRMSD) were determined with a Student’s *t*-test for each Cα position. The following criterion was used to assign regions with significant RMSD difference: *p*-value < 0.001 & |ΔRMSD| > 0.5 Å & *L* ≥ 3, where *L* is the number of consecutive positions satisfying the first two criteria.

### Normal mode analysis of PulG pilus models

We used the Anisotropic Network Model on Cα atoms in the program Prody (Bakan et al. 2011) to calculate normal modes of PulG pilus models, using a 15 Å distance cutoff for pairwise contacts. Global normal mode analysis (NMA) was performed on PulG pili models of 30 subunits, which we constructed from the helical symmetry of the 3D reconstruction and the central pilin coordinates from the real-space refined models. For each of the first three non-trivial lowest frequency modes, the average of the atomic mean square fluctuations was calculated for each subunit, excluding the first 24 residues. Local NMA was performed on a subset of atoms, included in a box expanding 15 Å outside the atomic coordinates of the central pilin from a pilus model along the Z-axis (i.e., pilus axis). The average of the atomic mean square fluctuations was calculated for the first three non-trivial lowest frequency modes. For local NMA, regions with significant difference in mean square fluctuations (Δfluctuations^2^) were determined with a Student’s *t*-test for each Cα position. The following criterion was used to assign regions with significant difference: *p*-value < 0.001 & Δfluctuations^2^ > σ & *L* ≥ 3, where σ is the average standard-deviation of mean square fluctuations and *L* is the number of consecutive positions satisfying the first two criteria.

### NMR analysis of differences in structure and dynamics of PulG monomers

Two samples of the periplasmic domain of PulG (25–134) wild type (PulG_WT_) and a mutant in which residues H106 and W129 were mutated to cysteine (PulG_CC_) were studied. The ^15^N relaxation times (T_1_ and T_2_) and {^1^H}–^15^N heteronuclear NOE were measured on each sample (0.15 mM in 50 mM HEPES, pH 7.0, 50 mM NaCl, 1 mM CaCl_2_) by standard methods (Barbato et al. 1992), in an interleaved manner. A recycling delay of 3 s, 8 scans and 7 relaxation delays were used for T_1_ (20, 140, 260, 500, 700, 1000, 1500 ms) and T_2_ (17, 34, 51, 68, 102, 136, 187 ms). The heteronuclear NOE were recorded in the presence and absence of a 2 s ^1^H saturation sequence (120° ^1^H pulse train), with a recycling delay of 5 s and 192 scans. The relaxation parameters were analyzed with the model-free formalism of Lipari and Szabo (1982) with the program TENSOR2 (Dosset et al. 2000) to extract internal dynamical parameters: order parameter S^2^ describing the amplitude of the motions; internal correlation time τ_e_ on the ps-ns time scale and R_ex_ reflecting exchange contribution on the μs–ms timescale. An anisotropic model with a diffusion tensor was necessary to describe the global reorientation of the PulG monomer, due to its elongated shape.

NMR experiments (2D ^1^H–^15^N HSQC and relaxation experiments) were performed at 25 °C on a 600 MHz Bruker Avance III spectrometer equipped with a TCI cryoprobe. The spectra were processed with NMRpipe (Delaglio et al. 1995) and analyzed with CcpNmr Analysis 2.4 software (Vranken et al. 2005).

Structural differences on PulG induced by the H106C and W129C mutations were estimated from chemical shift perturbations (Δδ^avge^), calculated as the weighted average (^1^H, ^15^N) chemical shift differences between PulG_WT_ and PulG_CC_ as follows: $\Delta \delta^{\text{avge}} = \left( {\left( {\Delta \delta \left( {^{1} {\text{H}}} \right)} \right)^{2} + \left( {\Delta \delta \left( {^{15} {\text{N}}} \right) \times 0.159} \right)^{2} } \right)^{1 /2}$.

### Analysis of solvent accessibility in the PulG monomer and the pilus by hydrogen/deuterium exchange mass spectrometry

A summary of the HDX-MS experiments is provided in Table S1. The quality of each protein was assessed prior to labeling by intact mass measurement (data not shown).

#### Sample preparation for HDX-MS

Deuterium exchange was initiated by adding 55 μL of deuterated buffer (50 mM HEPES, 50 mM NaCl, 2 mM CaCl_2_, pD 7.0) to 15 μL of PulG monomer (6 μM in 50 mM HEPES, 50 mM NaCl, 2 mM CaCl_2_, pH 7.0) or PulG pili (unknown concentration in the same buffer as PulG monomer). Prior to labeling, the concentration of the PulG pili was adjusted not to saturate the MS signal after labeling and quenching. Continuous labeling was performed at 20 °C for t = 0.16, 1, 10, 30, 60, 120 and 180 min. Aliquots of 10 μL were removed and quenched upon mixing with 50 μL of an ice cold solution of 3% formic acid, 4 M urea to decrease the pH to 2.5. Quenched samples were immediately snap frozen in liquid nitrogen and stored at − 80 °C until MS acquisition. Undeuterated PulG samples were obtained by following the same experimental procedure and further used to generate peptide maps. Fully labeled controls were prepared by mixing 15 μL of PulG samples with 55 μL of 50 mM HEPES, 50 mM NaCl, 2 mM CaCl_2_, 8 M urea-d4, pD 7.0, incubated 24 h at 20 °C and processed as described above. All samples were prepared in triplicate for each time point and condition (independent replicates).

#### Data acquisition

Quenched samples were thawed and immediately injected onto a nanoACQUITY UPLC M-Class system equipped with the HDX technology (Waters Corporation, Milford, MA) and maintained at 0 °C to minimize back-exchange. Labeled samples (10.7 pmol PulG monomer, unknown quantity for PulG pili) were digested on an in-house pack column of immobilized pepsin (2.0 × 20 mm, 66 μL bed volume) for 2 min at 20 °C. Generated peptides were immediately trapped and concentrated onto a C18 Trap column (VanGuard BEH 1.7 μm, 2.1 × 5 mm, Waters Corporation, Milford, MA) at a flow rate of 100 μL/min (0.15% formic acid) and separated by a 7-min linear gradient of 5–40% acetonitrile at 40 μL/min using an ACQUITY UPLC BEH C18 analytical column (1.7 µm, 1 × 100 mm, Waters Corporation, Milford, MA). After each run, the pepsin column was manually cleaned with two consecutive injections of 1% formic acid, 5% acetonitrile, 1.5 M guanidinium chloride, pH 1.7. Blank injections were performed between each run to confirm the absence of carry-over. Mass spectra were acquired on a Synapt G2-Si HDMS mass spectrometer (Waters Corporation, Milford, MA) equipped with a standard ESI source and lockmass correction. Peptides were identified in undeuterated samples by a combination of data independent acquisition (MS^E^, ramp trap collision energy from 15 to 45 V) and exact mass measurement (below 5.0 ppm mass error) using the same chromatographic conditions than for the deuterated samples.

#### Data analysis

Peptide maps were database searched in ProteinLynX Global server 3.0 (Waters corporation, Milford, MA) with the following processing and workflow parameters: low and elevated intensity thresholds set to 100.0 and 50.0 counts; intensity threshold sets to 750.0 counts; variable modification: N-terminal methylation; non-specific primary digest reagent; false discovery rate set to 4%. Each fragmentation spectrum was manually inspected for assignment confirmation. The peptide map was refined in DynamX 3.0 (Waters corporation, Milford, MA) with a minimum product per amino-acid value of 0.4. DynamX 3.0 was used to extract the centroid masses; only one unique charge state was considered per peptide and no back-exchange correction was performed. HDX results are reported as relative deuterium exchange level expressed in either mass unit or fractional exchange. Fractional exchange data were calculated by dividing the experimental uptake value by the theoretically maximum number of exchangeable backbone amide hydrogens that could be replaced into each peptide in 78.6% excess deuterium. MEMHDX (Hourdel et al. 2016) was used to visualize and statistically validate the HDX data (Wald test, *p*-value < 0.01).

## Results

### Refined pseudo-atomic model of PulG_WT_

The cryo-EM map that had initially been obtained of the wild type form of the PulG pilus was limited to about 7.5 Å resolution (López-Castilla et al. 2017), due to the high degree of flexibility of the whole system. The NMR structure of the truncated PulG pilin and the detailed characterization of its dynamics highlight flexibility in the ps-ns timescale in the β1/β2 loop and in the C-terminus (López-Castilla et al. 2017 and see below). To stabilize this region an aromatic stacking interaction between side-chains of H106 and W129 was replaced by an intra-molecular disulfide bond (mutant PulG_CC_, H106C/W129C). Indeed, the EM maps obtained with PulG_CC_ pili were of higher resolution (around 5 Å) and allowed us to obtain a pseudo-atomic model. Paradoxically, however, the PulG_CC_ pili were less stable, and disassembled more readily compared to the native fibers (López-Castilla et al. 2017).

**Fig. 1:**
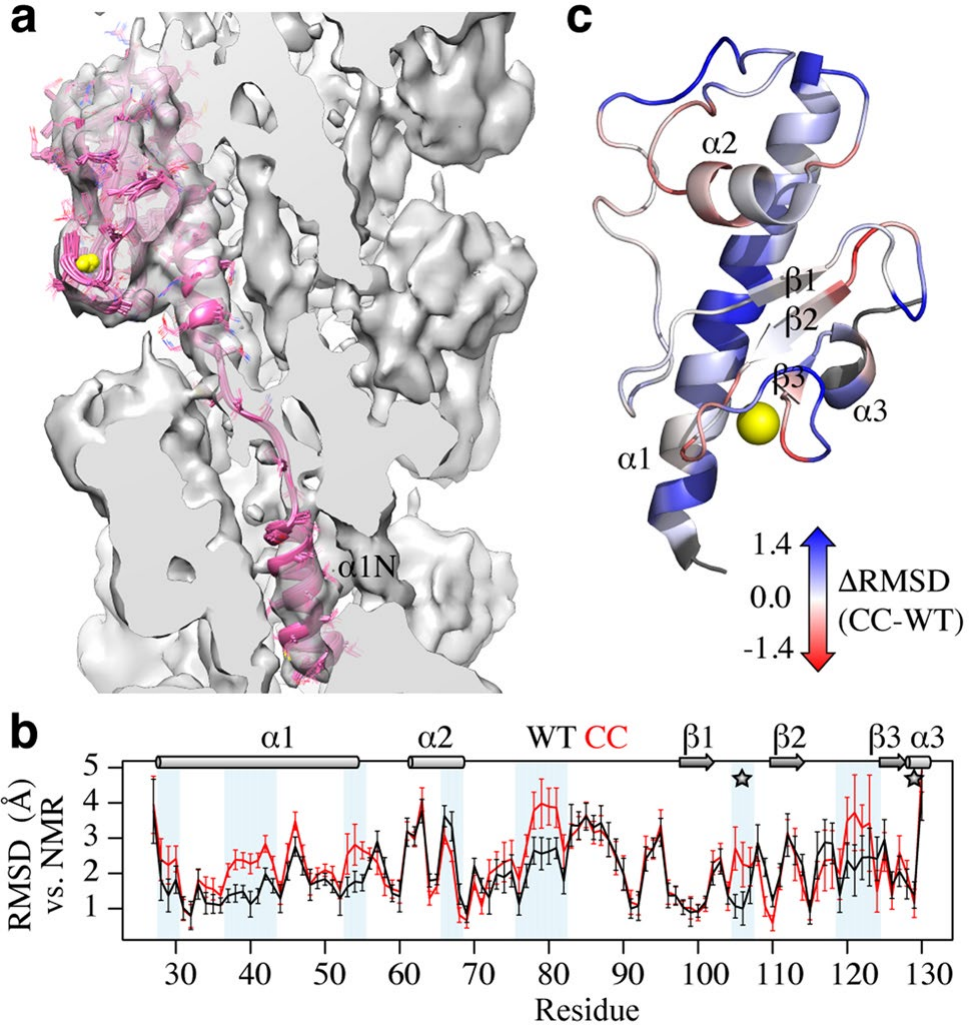
Refined PulG pili models. **a** Cross-section of the PulG_WT_ cryo-EM reconstruction at ~ 7.5Å  resolution with a single PulG subunit from the refined ensemble. **b** RMSD along the PulG sequence between PulG_WT_ NMR structure and PulG_WT_ pili (black) or PulG_CC_ pili (red) models. Regions with significant RMSD difference (ΔRMSD) are highlighted in blue. The mutation positions H106C and W129C are indicated by gray stars. ** c** Mapping of ΔRMSD on the PulG_WT_ structure. As indicated by the color coding, blue corresponds to “PulG_WT_ is closer to NMR” and red means “PulG_CC_ is closer to NMR”, white, no difference

In order to build three-dimensional models of the PulG_WT_ pilus, we used the structure of the PulG_CC_ pilus as an initial template for model construction and real-space refinement in the PulG_WT_ cryo-EM reconstruction. To account for the lower resolution of the PulG_WT_ cryo-EM reconstruction compared to the PulG_CC_ reconstruction, and not rely on a single 3D model, we calculated a series of 11 models for both PulG_WT_ and PulG_CC_ pili after refinement in their respective EM density maps starting from perturbed initial models. Ensembles of refined models displayed good convergence with average backbone RMSDs to the mean of 0.47 ± 0.09 Å and 0.48 ± 0.10 Å for PulG_WT_ and PulG_CC_ pili, respectively. Additionally, the fit of the refined models to their respective cryo-EM density was similar with cross-correlation coefficients of ~ 0.78 (Table S2). Ensembles of refined models in the cryo-EM density are shown in Fig. 1a and S1. The PulG_WT_ and PulG_CC_ models are very similar but exhibit local structural differences, as illustrated by the relatively small backbone RMSD between them (1.20 ± 0.13 Å for the central subunit, Fig. S1). Each model was compared to the solution NMR structure of PulG_WT_ (PDB 5O2Y). Since the PulG_WT_ soluble domain does not contain the α1-N helix, the comparison was restricted to residues 27–130. The PulG_WT_ pilus model is closer to the solution PulG_WT_ structure than the PulG_CC_ pilus model, with average backbone RMSDs of 2.15 ± 0.06 Å and 2.41 ± 0.09 Å, respectively (Fig. 1b). Superimposed structures of soluble PulG_WT_ and refined PulG_WT_ and PulG_CC_ pili are shown in Fig. S1. Most of the regions displaying significant differences in RMSD correspond to regions where the PulG_WT_ pili model is structurally closer to the solution NMR structure (Fig. 1c). These regions include most of the α1 helix, the apical part of the α2/β1 loop, part of the calcium binding loop and the tip of the β1/β2 loop, around H106. Conversely, the C-terminal part of the α2 helix is closer to the solution conformation of PulG in the PulG_CC_ models.

### Overall dynamics of PulG pili

**Fig. 2:**
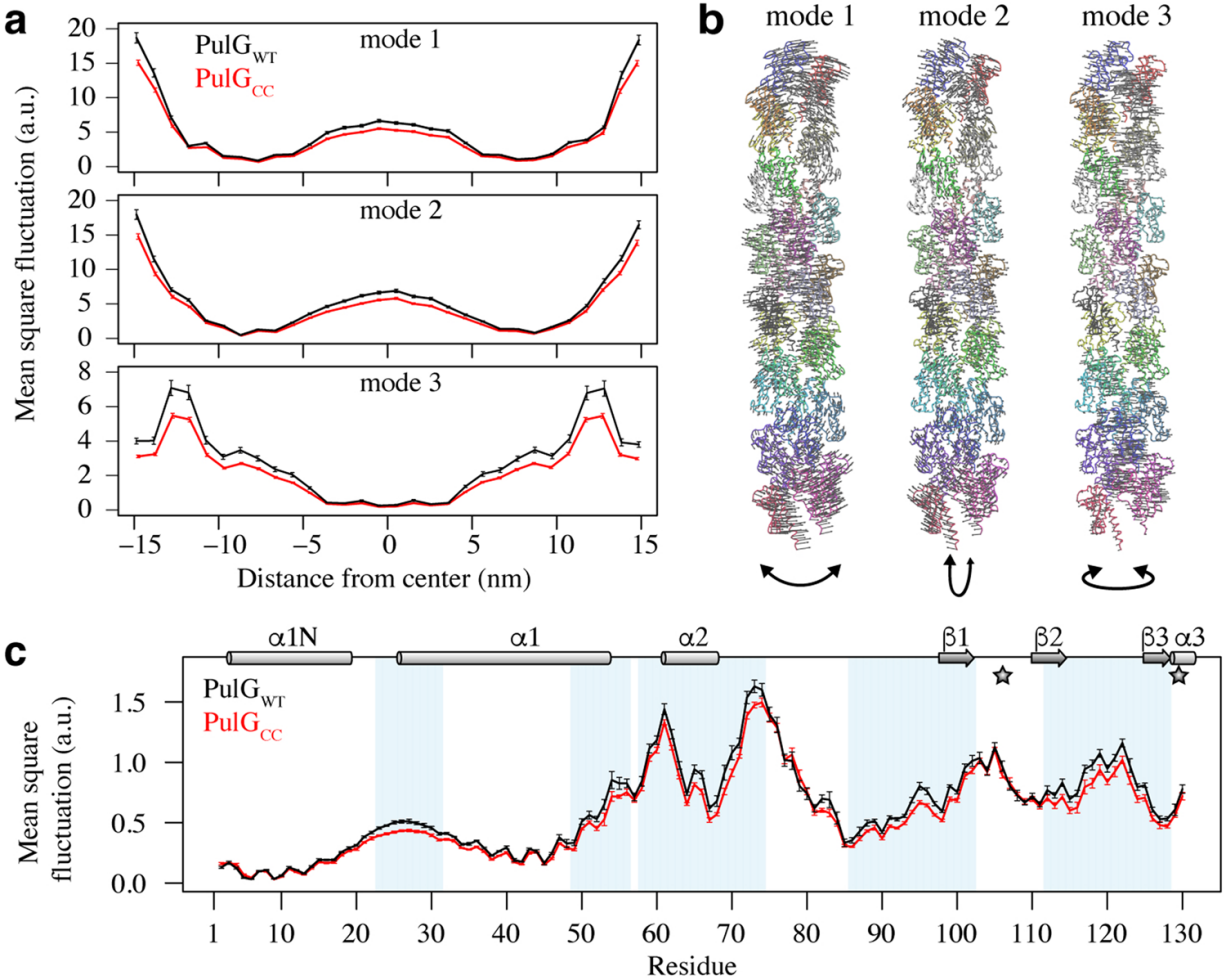
Normal mode analysis of PulG pili models. **a** Average of the mean square fluctuations per subunit (global) along the pilus axis for each first three non-trivial low frequency modes in PulG_WT_ (black) and PulG_CC_ (red) pili models. **b** Representations of the direction of individual modes per atom (arrows) on the PulG pilus structure, colored by subunit. For each mode, the overall movement is represented by a curved arrow below the pilus structure. **c** Average of the mean square fluctuations per residue (local) along the PulG sequence using the first three non-trivial low frequency modes in PulG_WT_ (black) and PulG_CC_ (red) pili models. Regions with significant difference in mean square fluctuations are highlighted in blue. The mutation positions H106C and W129C are indicated by gray stars

To obtain information on the dynamic behavior of PulG_WT_ and PulG_CC_ pili models, we employed normal mode analysis (NMA). We predicted global and local mobility using either long pili models (30 subunits, ~ 30 nm long) or slices of the pili models, along the pili axis, encompassing at least one complete subunit. Globally, the first three non-trivial modes show similar dynamic behavior for both PulG_WT_ and PulG_CC_ pili (Fig. 2a). Modes 1 and 2 represent bending movements of the pili in two perpendicular directions. Movements associated with mode 3 correspond to a twisting of the pili around their helical axes (Fig. 2b). The largest mean square fluctuations are observed for subunits at the extremities of the pili, and at the center for bending modes. For each mode, PulG_WT_ models display larger mean square fluctuations than PulG_CC_ models. Local NMA performed on pili slices yielded similar behavior as full pili, where the first three lowest frequency modes correspond to bending and twisting movement (except that in that case, the twisting movement describes the slowest mode). Profiles of atomic fluctuation amplitudes along the PulG sequence for the central subunit are highly similar for PulG_WT_ and PulG_CC_ pili (Fig. 2c). As expected, the predicted mobility for regions without secondary structure are higher, i.e., the unfolded region at the end of α1-N, the α1/α2 loop, the apical part of the α2/β1 loop, the β1/β2 loop and the calcium binding loop. PulG_WT_ pili display significantly larger mobility, especially in the soluble domain of PulG (residues 20–130), even though differences in fluctuations for PulG_WT_ and PulG_CC_ pili are subtle. Our explorations of global and local dynamics of pili by NMA reveal that small motions in the pilin subunit can be propagated and amplified in fully assembled pili, owing to the elongated and repetitive nature of such a filament.

### Detailed analysis of the dynamics of individual PulG monomers

To get a more detailed picture of the influence of the local dynamics of the pilins on pilus assembly and stability, we characterized and compared the dynamics of PulG_WT_ and PulG_CC_ monomers by solution NMR.

The ^1^H and ^15^N backbone resonances of PulG_CC_ could be readily assigned by homology to their chemical shift in the wild type form. The differences in chemical shift are very localized around the mutation site (Fig. S2), indicating that the structural changes introduced by the mutation are also very local. The backbone dynamics of both forms of PulG monomers were studied by measuring ^15^N NMR relaxation parameters (T_1_, T_2_ and heteronuclear NOE), which are very sensitive to overall and internal motions. The relaxation parameters were analyzed with an anisotropic diffusion tensor to describe the global reorientation, and the Lipari–Szabo model-free approach (Lipari and Szabo 1982) was used to extract the internal dynamic parameters (S^2^, τ_e_ and R_ex_).

**Fig. 3:**
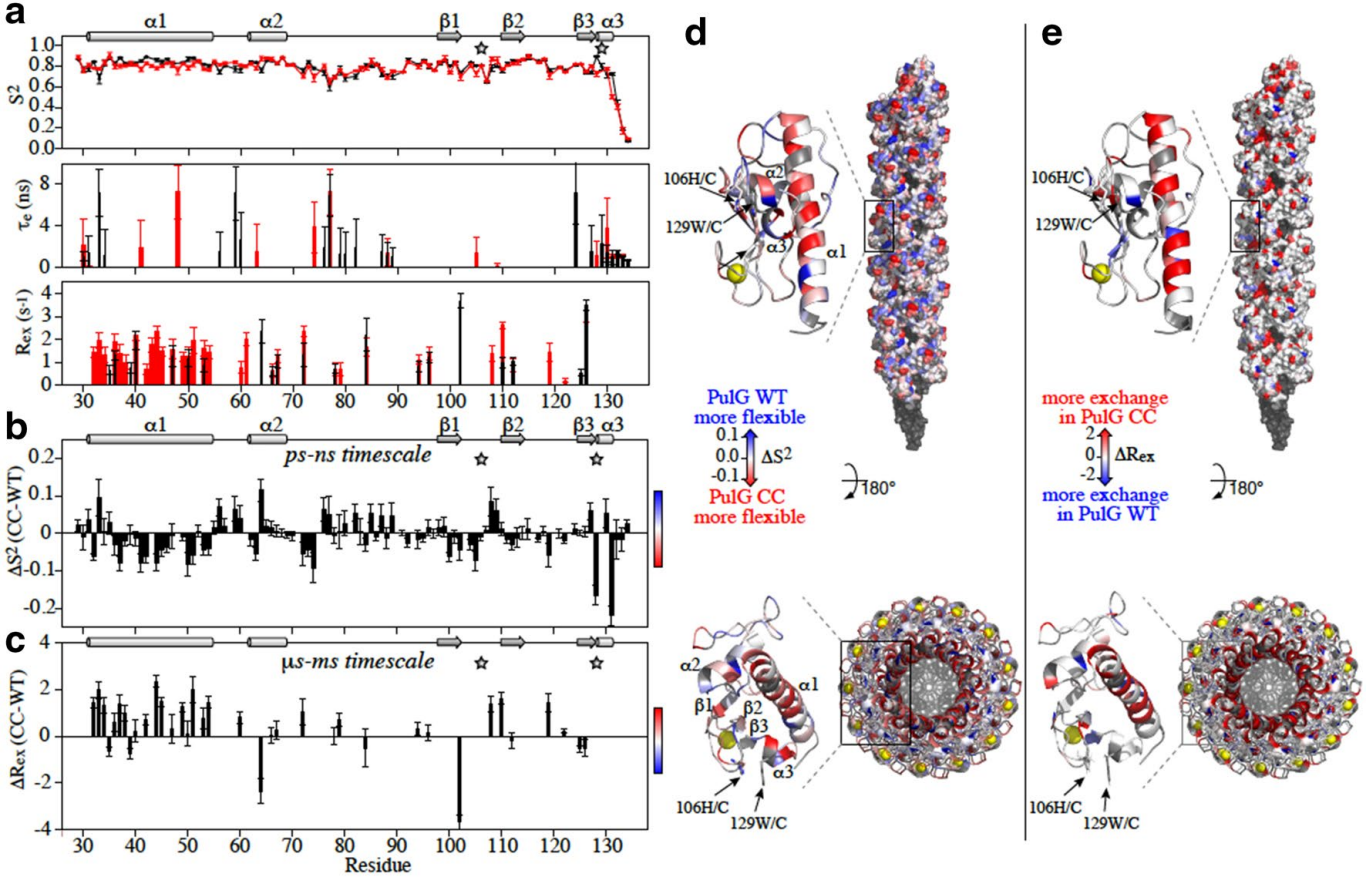
Dynamic changes on PulG monomer induced by the H106C-W129C mutation. **a** Dynamic parameters extracted from the ^15^N relaxation data at 600 MHz using the model-free formalism of Lipari-Szabo with an anisotropic global reorientation model: amplitude of the picosecond (ps) to nanosecond (ns) time scale motion (S^2^), internal correlation time (τ_e_) and exchange contributions on the μs-ms timescale (R_ex_). The mutation positions H106C and W129C are indicated by gray stars. **b**, **d** Variation of S^2^ ($\Delta S_{{({\text{CC}} - {\text{WT}})}}^{2}$) highlighting ps–ns time scale dynamics and its mapping on the PulG monomer and pilus structure, side and top view. **c**, **e** Variation of R_ex_ (ΔR_ex(CC-WT)_) reflecting μs–ms timescale dynamics and its mapping on the PulG monomer and pilus structure. Blue and red indicate less or more flexibility in the mutant, respectively. The calcium atoms are displayed in yellow, only on the ribbon representation

The diffusion tensors are very similar for PulG_WT_ and PulG_CC_ with amplitudes D_x_, D_y_, D_z_ of (1.95 ± 0.01, 2.15 ± 0.02, 2.88 ± 0.02) × 10^7^ s^−1^ and (2.05 ± 0.02, 2.16 ± 0.02, 2.66 ± 0.02) × 10^7^ s^−1^, respectively, indicating similar anisotropic overall tumbling. PulG_WT_ and PulG_CC_ display similar profiles of T_1_, T_2_ and NOE, leading to comparable internal dynamics behavior at a first glance (Fig. 3a). In both forms, the secondary structures are rather rigid on the ps–ns timescale, showing small amplitudes of motion indicated by order parameters, S^2^, close to 0.8. The most flexible regions (S^2^ < 0.7) are localized in the portions of loops that are not tightly packed against the core of the protein (e.g., H76-G83 and R87-Q91 in loop α2/β1, and in loops β1/β2 and β2/β3 of the region surrounding the calcium-binding site) and in the C-terminus (T130-K134). However, changes are observed in ps-ns timescale dynamics between PulG_WT_ and PulG_CC_ from the difference in S^2^ ($\Delta S_{{({\text{CC}} - {\text{WT}})}}^{2}$, Fig. 3b, d). The mutation induces long-range effects, such as a reduction of the amplitudes of fast motion ($\Delta S_{{({\text{CC}} - {\text{WT}})}}^{2} > 0$) in some loops, mainly α1/α2 and β1/β2. These regions are solvent exposed and at the outside of the pilus (Fig. 3d). Conversely, the mutation increases flexibility all along the α1 helix ($\Delta S_{{({\text{CC}} - {\text{WT}})}}^{2} < 0$). This helix undergoes also the most pronounced changes of μs–ms timescale dynamics, with more residues of the mutant displaying relaxation parameters (ΔR_ex__(CC-WT)_ > 0) indicative of conformational exchange (Fig. 3c, e).

### Solvent accessibility of PulG in isolation and in the pilus

HDX-MS is the experiment of choice to characterize the structure and dynamics of large macromolecular complexes and changes occurring upon their formation. Backbone amide hydrogens fully exposed to the solvent and not involved in secondary structural elements exchange very rapidly whereas those located in secondary structural elements exchange at much slower rates due to hydrogen bonding (Wales and Engen 2006). We used HDX-MS to probe and compare the solvent accessibility of PulG_WT_ in the monomer and the pilus in order to understand the changes induced by the formation of the pilus, and further validate the placement of the N-terminal helix in the pilus. Regions of PulG_WT_ buried within the pilus or involved in subunit:subunit interactions are expected to be less accessible to the solvent compared to the monomer.

**Fig. 4:**
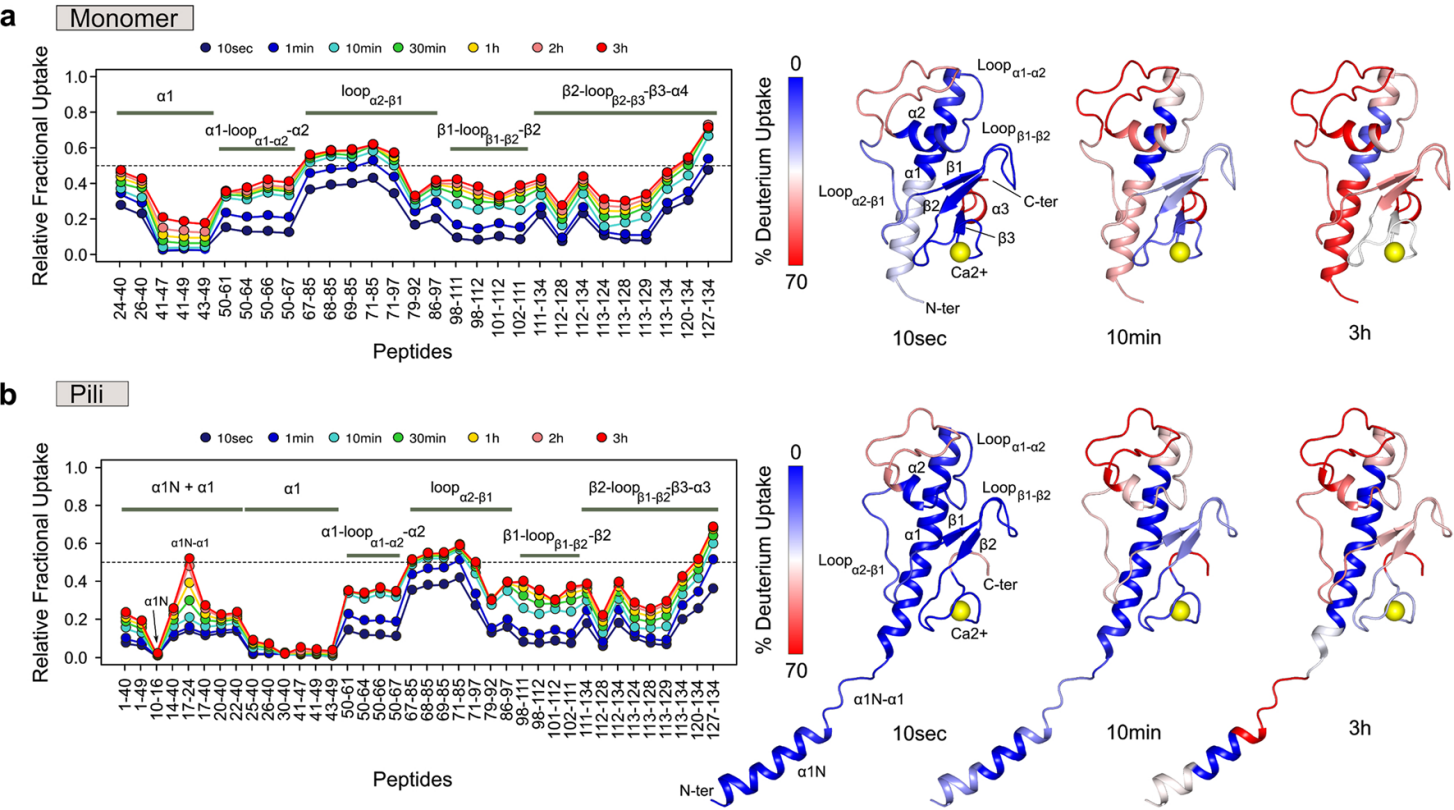
Deuterium exchange profiles of PulG_WT_ in the monomer (**a**) and in the pilus (**b**) measured at 20 °C and pD 7.0. Relative fractional exchange values were determined at each time point and plotted as a function of peptide position (from N- to C-terminal) using MEMHDX. Each dot corresponds to an average of three independent HDX-MS experiments. The solvent accessibility measured after 10 s, 10 min and 3 h incubation is mapped onto the NMR structure of PulG_WT_ monomer (**a**) and the model (**b**) of PulG_WT_ pilus from the cryo-EM reconstruction

**Fig. 5:**
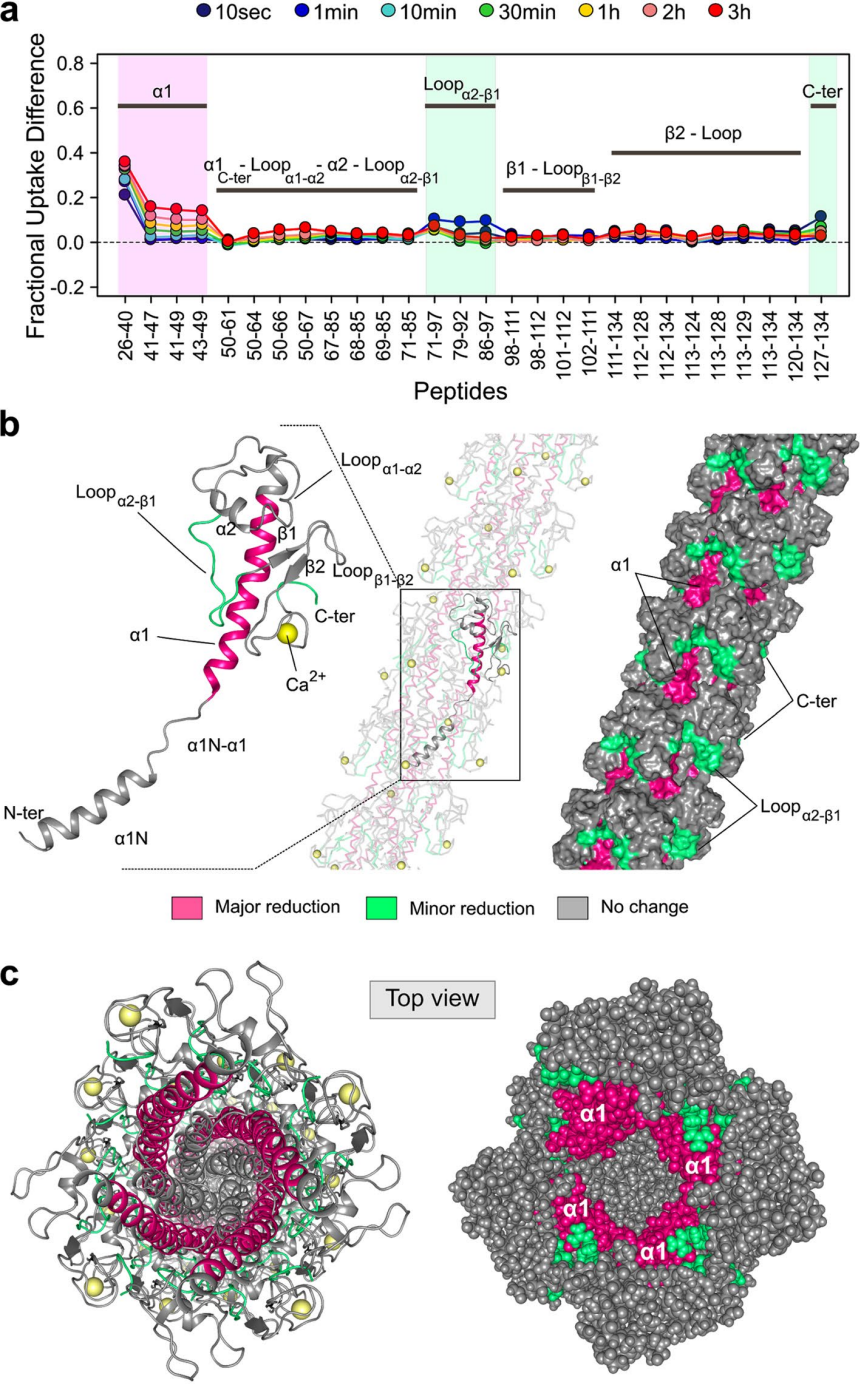
Difference of solvent accessibility between the PulG_WT_ monomer and the pilus. **a** Fractional deuterium uptake difference plot showing the difference in deuterium incorporation between PulG_WT_ monomer and PulG_WT_ in the pilus at each time point, and for each identical peptide. A positive value indicates a reduction of solvent accessibility of PulG_WT_ in the pilus compared to the monomer. The three regions of PulG_WT_ showing statistically significant reduction of solvent accessibility (Wald test, *p *< 0.01) are mapped onto the PulG subunit and the cryo-EM pilus model (**b**, **c**). The threshold between major (pink) and minor (green) reductions of deuterium uptake was set to 10% (major reductions > 10%; minor reductions > 5% and below 10%; no change of solvent accessibility < 5%)

The two protein samples of the monomeric pilin and the pilus were digested with pepsin in the presence of urea to generate a set of overlapping peptides covering 100% of each protein sequence (Fig. S3). The exchange behaviors of the monomer and the pilus were further visualized by plotting each calculated relative fractional uptake value as a function of peptide position (Fig. 4, S4). Dynamic HDX-MS activities (i.e., increase of deuterium uptake over the labeling time course), indicative of the presence of secondary structural elements, were observed throughout both protein states, confirming that the monomer and the pilus were well folded. The difference in relative fractional uptake reveals that regions 50–85 and 98–126 display identical deuterium incorporation rates in the monomer and the pilus (Fig. 5a, b). This result provides direct evidence that, in the context of the pilus, the α1-C (residues 50–54) and α2 helices, the β1–β2–β3 sheet, and the loops connecting α1–α2, α2–β1 (residues 69–85), β1–β2 and β2–β3 remain accessible, and are therefore neither buried nor involved in subunit:subunit interactions. Regions 26–49 (α1), 86–97 (loop α2/β1) and 127–134 (C-terminal peptide), on the other hand, display statistically significant reduction in deuterium uptake in the pilus compared to the monomer (Fig. 5a, b). The major reductions of solvent accessibility are observed in peptides 26–40, 41–47 and 43–49 covering the entire α1 helix. This change of accessibility is in line with our cryo-EM structure and the position of α1 in the central core of the fiber (Fig. 5c). In addition, the α1 helix contains two important negatively charged residues (E44 and D48) involved in conserved inter-subunit contacts with the positively charged residues R87 and R88 (López-Castilla et al. 2017). Consequently, the slight reduction of solvent accessibility observed in segment 86–97 results from direct inter-subunit interactions. Finally, the C-terminal peptide 127–134 does not appear to be buried in our cryo-EM structure or to be involved in inter-subunit interactions. The observed reduction of solvent accessibility suggests that region 127–134 adopts a more stable conformation in the fiber than in the monomer.

The monomer lacks α1-N, which limits the comparison of solvent accessibility with the pilus to residues 24–134. However, the exchange profiles of peptides covering residues 1–23 contain informative structural data. As observed with α1, the central region of α1-N (peptide 10–16) is completely buried in the fiber, explaining the absence of deuterium uptake (Figs. 4b, 5). Peptide 17–24, on the other hand, appears well exposed to the solvent and reaches a final incorporation level similar to loop α2/β1 (residues 68–71). The exchange behavior of peptide 17–24 fits nicely with the proposed extended non-helical structure connecting α1-N with the C-terminal half of α1 and indicates that, in the context of the fiber, this region is accessible to the solvent (Fig. 4b). A similar result was observed by HDX-MS on the *Vibrio cholerae* Toxin-coregulated pilus (Li et al. 2008).

## Discussion

In this paper we obtained a consistent picture, at an atomistic scale, of the structure and dynamics of a large, polymeric system, a Type 2 Secretion System pseudopilus. In particular, we gained understanding of how local dynamics is implicated in the assembly and stability of the pilus, and how small structural and dynamics differences on the local scale can have long range effects on the behavior of the whole system. For this, we used data from NMR, cryo-EM and HDX-MS, in combination with molecular modeling and normal mode analysis, to obtain a consistent description of the dynamics of the pilus at several time and length scales, covering times from ps–ns to µs–ms (by NMR), and minutes to hours (by HDX-MS).

NMR has been instrumental in this integrative structural biology project at several points. It was used to determine the high resolution structure of the pilus subunit PulG. The structural and dynamic behavior of PulG led to the design of a mutant that resulted in an EM map of much higher resolution for the pilus. The detailed analysis of differences in the dynamic behavior between wild type and mutant PulG may in turn provides an explanation of the surprising fact that the introduced mutations increased order of the pilus, improving the resolution of its EM reconstruction, yet the mutant pilus was observed to be more fragile than the wild type (López-Castilla et al. 2017). Although the differences in internal dynamics between the two forms of PulG are small (ΔS^2^ < 0.1 and ΔR_ex_ < 2), they could have a cumulative effect on inter-subunit interactions along the fiber. The decrease of ps–ns time scale dynamics in loops of the mutant that are solvent exposed and at the outside of the pilus could explain the gain in resolution in cryo-EM. Conversely, the mutation and the constraint of the disulfide bridge at the C-terminus of the protein seem to be compensated by increased dynamics in both fast and slow timescales at the N-terminus and particularly in the α1 helix, which is packed at the core of the pilus (Fig. 3d, e). This dynamic behavior might explain the lower stability of the mutant pilus in vivo (López-Castilla et al. 2017).

Remarkably, the reduced overall flexibility of the mutant pilus is captured by a simple normal mode analysis. NMR, NMA and HDX-MS give a consistent picture of the PulG pilus as a large, ordered but flexible system. These approaches are highly complementary in the characterization of the system under study. NMR is used to obtain complete and detailed analysis of the internal dynamics of individual subunits, with the amplitude and frequency of the motion. NMA provides the global picture of the overall, correlated movements of the entire system. HDX-MS informs on the molecular assembly and protein–protein contacts.

HDX-MS also provides detailed information on dynamic behavior of large assemblies that cannot be easily gained from a rigid structure alone. A case in point is the region of increased exchange in the long N-terminal helix, which perfectly maps onto the location of the helix discontinuity, a region that could not be readily assigned based on the medium-resolution wild type EM map alone. The results clearly show that the region of the helix discontinuity is accessible despite the fact that it is in the interior of the pilus, again underlining the flexibility of the system. The results also show that the helix discontinuity is not a result of sample preparation for EM, which puts some strain on the fibers. Our HDX results are very similar to those obtained for the T4 *Vibrio cholerae* Toxin-coregulated pilus (Li et al. 2008). This suggests that the helix discontinuity is a conserved feature of T2SS and T4 pili, including those with more diverging sequences such as the *Vibrio cholerae* Toxin-coregulated pilus. This conserved structural feature of the N-terminal helix provides flexibility, crucial for the dynamic assembly and function of the whole pilus.

## Electronic supplementary material

Below is the link to the electronic supplementary material.

Supplementary material 1 (DOCX 2697 kb)
