# Supplementary material for: Dynamics of a type 2 secretion system pseudopilus unraveled by complementary approaches
Source: J Biomol NMR. 2019 May 23;73(6):293–303. doi: 10.1007/s10858-019-00246-4 (PMC6692295; doi:10.1007/s10858-019-00246-4)
Supplement: Supplementary file 1 — Supplementary material 1 (DOCX 2697 kb) [file 10858_2019_246_MOESM1_ESM.docx]

**Supplementary Materials**

**Dynamics of a type 2 secretion system pseudopilus unraveled by complementary approaches**

**Benjamin Bardiaux,^1^ Florence Cordier,^1,2^ Sébastien Brier,^2^ Aracelys López-Castilla,^1^ Nadia Izadi-Pruneyre,^1*^ Michael Nilges^1*^**

1: Structural Bioinformatics Unit, Department of Structural Biology and Chemistry, C3BI, Institut Pasteur; CNRS UMR3528; CNRS USR3756; Paris, France

2: Biological NMR Technological Platform, Center for Technological Resources and Research, Department of Structural Biology and Chemistry, Institut Pasteur; CNRS UMR3528; Paris, France

correspondance to: [nadia.izadi@pasteur.fr](mailto:nadia.izadi@pasteur.fr), [michael.nilges@pasteur.fr](mailto:michael.nilges@pasteur.fr)


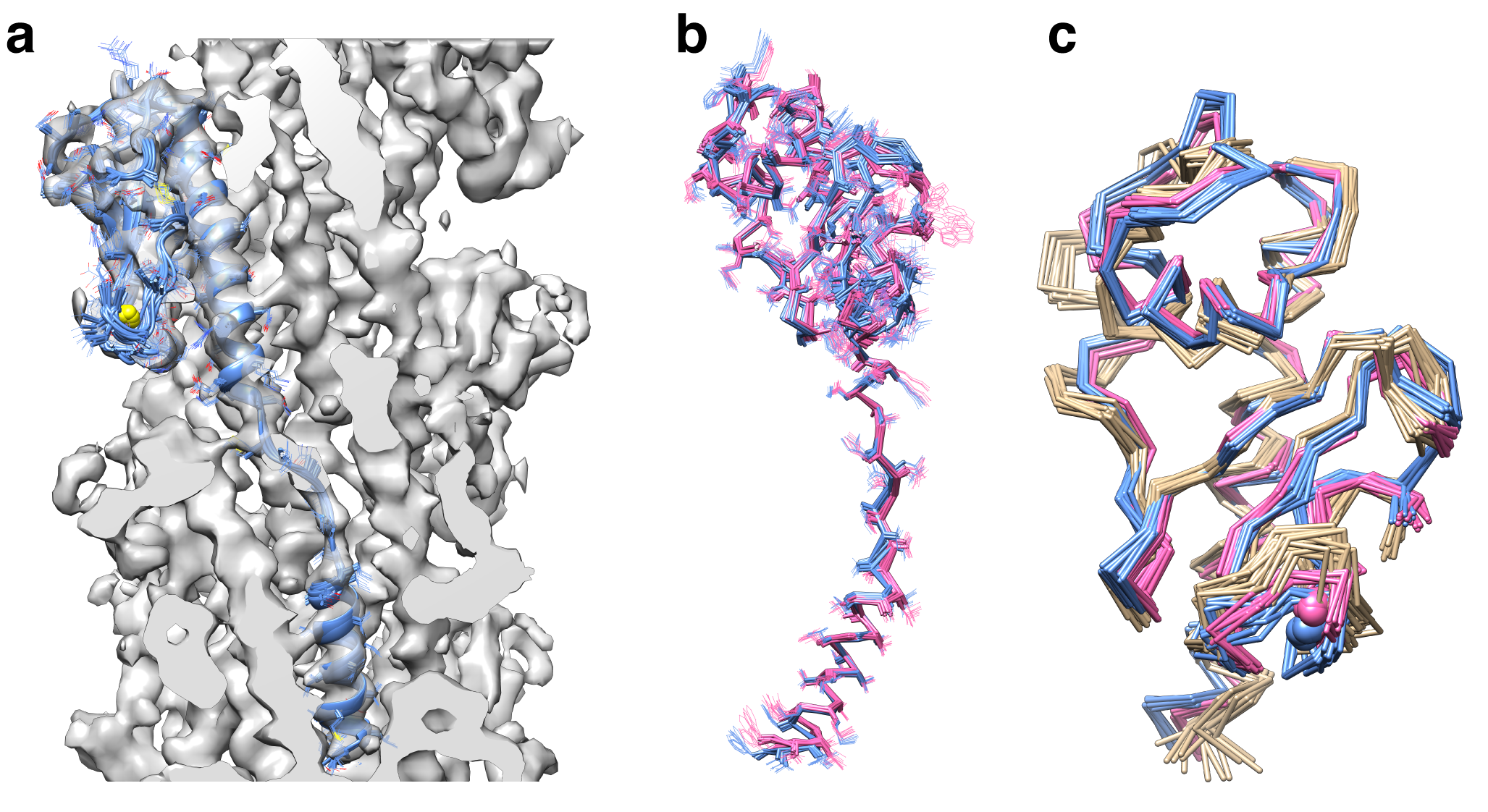


**Figure S1: Superimposed structures of soluble PulG_WT_ and refined PulG_WT_ and PulG_CC_ pili.** (a) Cross-section of PulG_CC_ cryo-EM reconstruction at 5 Å, with a single PulG subunit from the refined ensembles. (b) Superimposition of PulG_WT_ (pink) and PulG_CC_ (blue) refined models. (c) Superimposition of solution NMR PulG_WT_ structure (beige), PulG_WT_ pili (pink) and PulG_CC_ pili (blue) models.


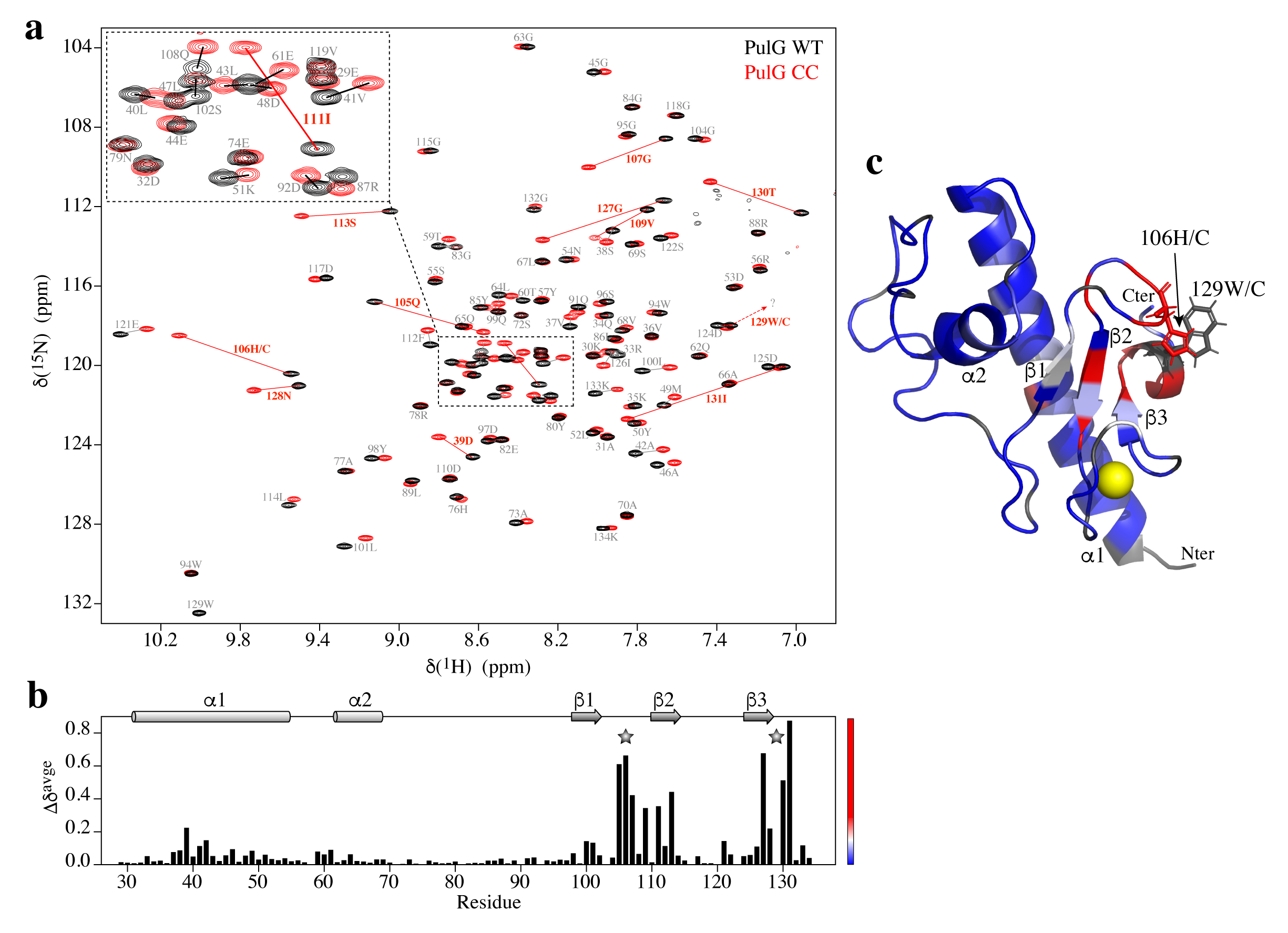


**Figure S2:** **Local effect of the H106C-W129C mutation on PulG monomer structure.** (A) Superimposed ^1^H-^15^N HSQC spectra of PulG_WT_ (black) and PulG_CC_ (red). The largest chemical shift differences are highlighted in red. (B) Weighted average ^1^H-^15^N chemical shift differences (Δδ^avge^) between PulG_WT_ and PulG_CC_. The mutation positions H106C and W129C are indicated by gray stars. Secondary structure elements are indicated on the top. (C) Mapping of mutation-induced chemical shift perturbations on PulG monomer structure, from blue to red with increasing Δδ^avge^. The calcium atom is displayed in yellow.

**
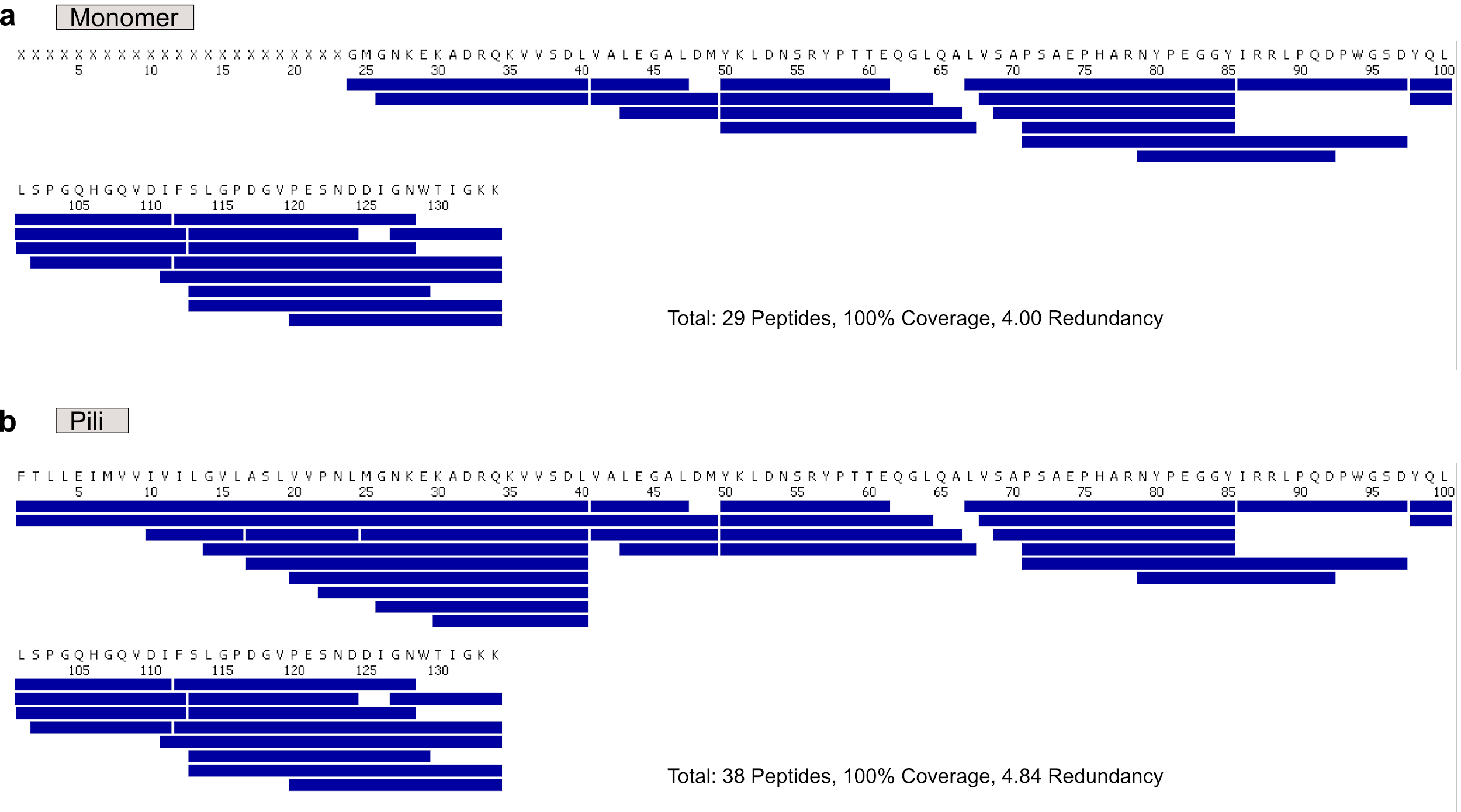
**

**Figure S3**: **Peptide maps of PulG_WT_ monomer (a) and PulG_WT_ pilus (b) generated after 2 min pepsin digestion at 20ºC and pH 2.5**. Each blue bar corresponds to a unique peptide. A 100% sequence coverage was achieved for both proteins. Missing residues in the N-terminal region of the PulG_WT_ monomer have been replaced by “X”.


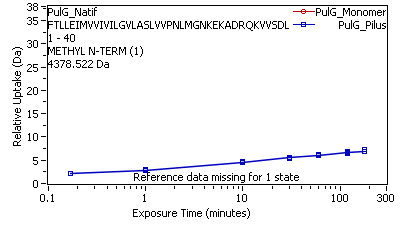

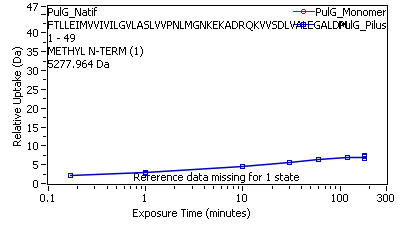

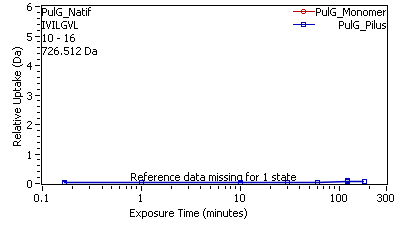


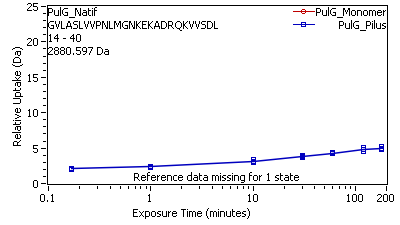

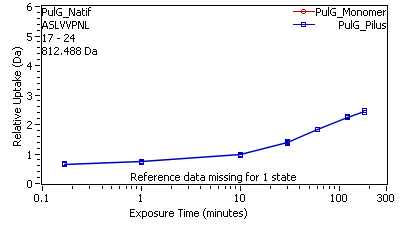

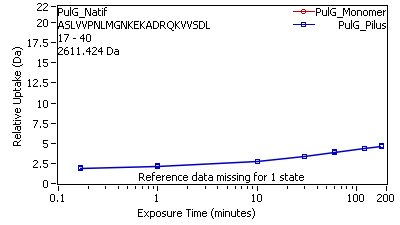


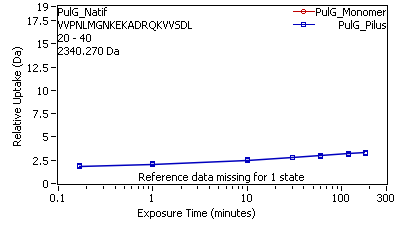

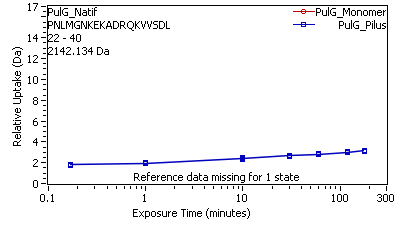

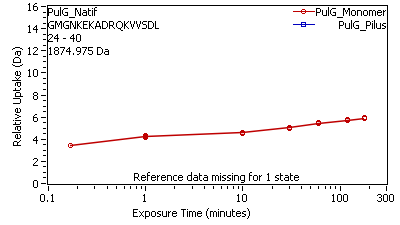


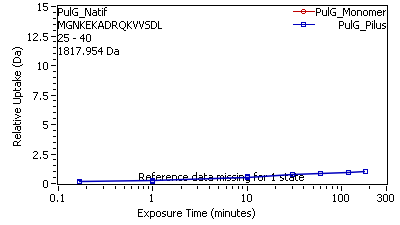

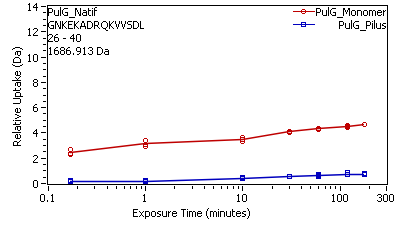

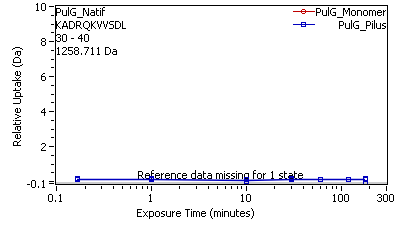


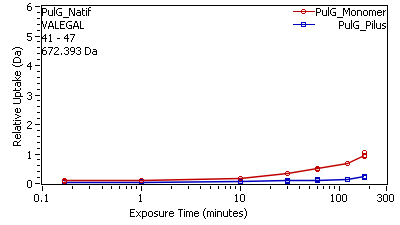

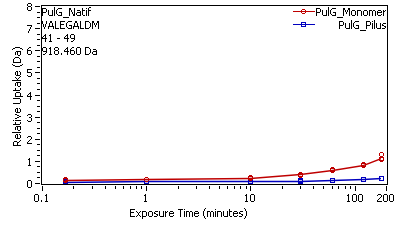

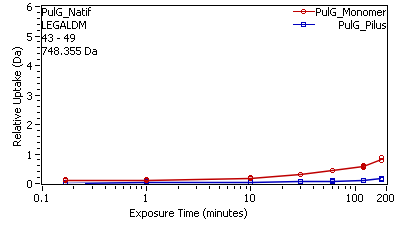


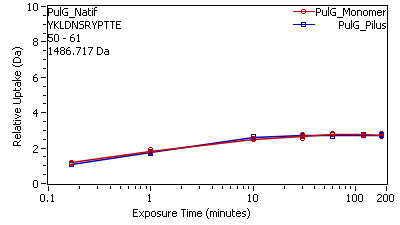

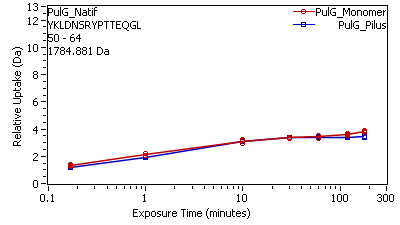

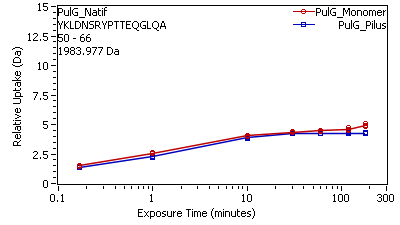


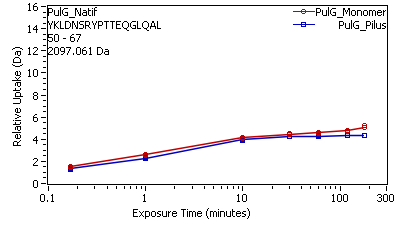

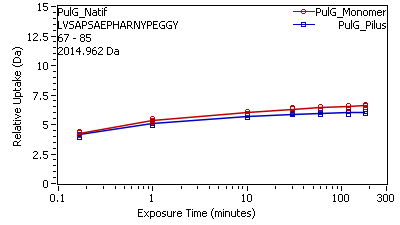

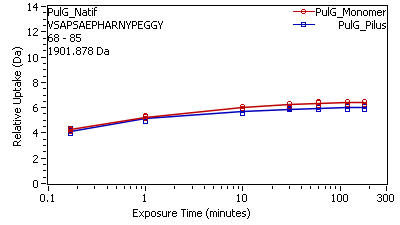


**Figure S4:** **Deuterium uptake curves for all peptides of PulG_WT_ analyzed in the monomer (red dots) and in the pilus (blue squares).**


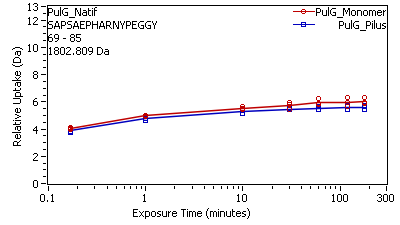

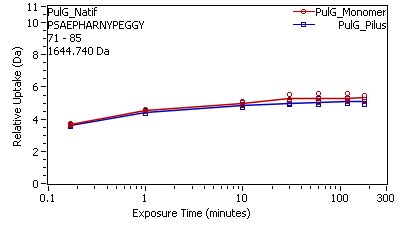

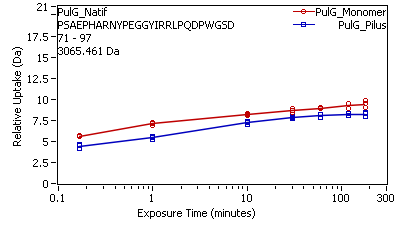


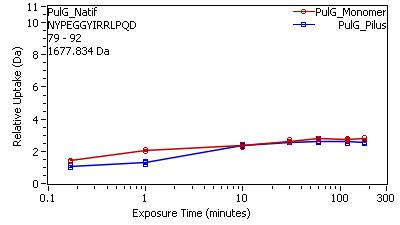

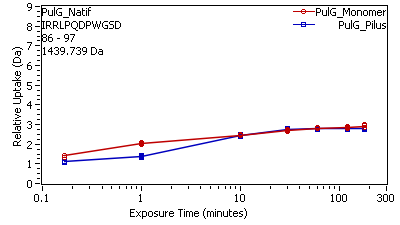

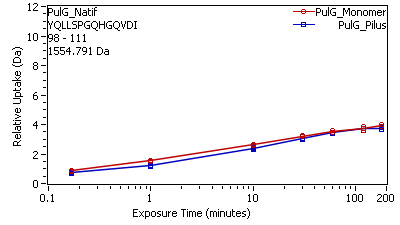


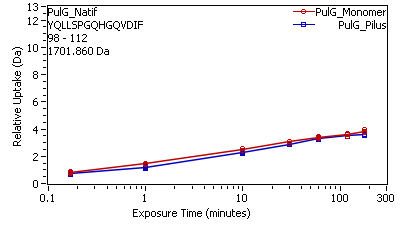

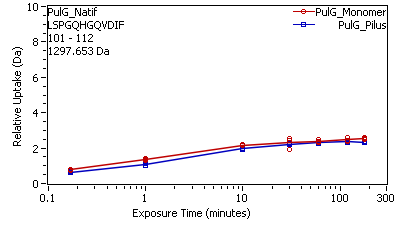

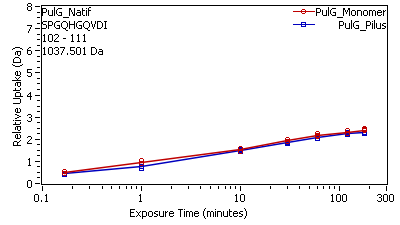


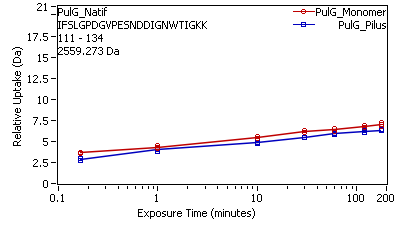

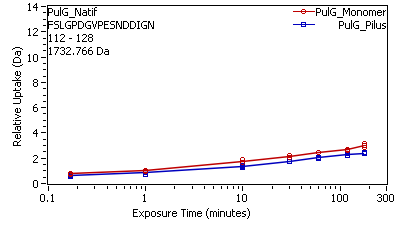

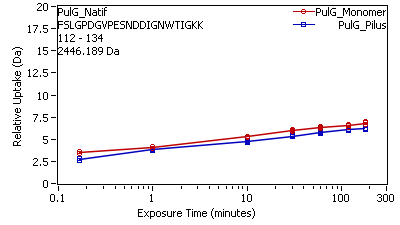


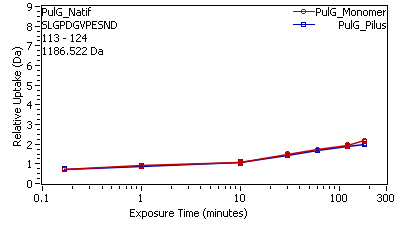

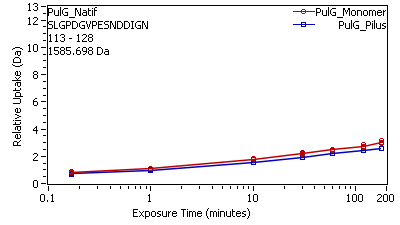

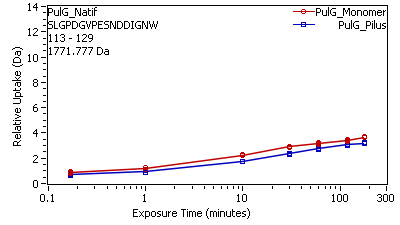


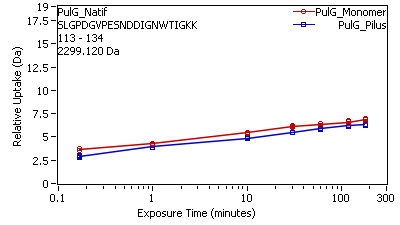

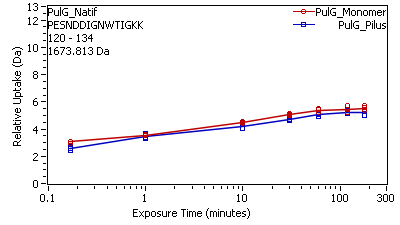

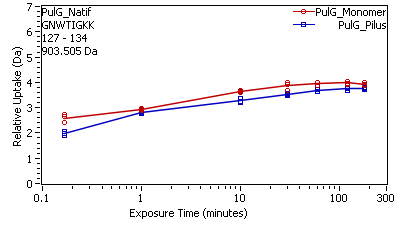


**Fig. S4:** **continued**

**Table S1: Summary of the Hydrogen/Deuterium eXchange – Mass Spectrometry (HDX-MS) experiments**

| **EXPERIMENT** | **PulG_WT_** **monomer** | **PulG_WT_ pili** |
| --- | --- | --- |
| HDX reaction details  *pD:*  *T°C:*  *Excess deuterium:* | 7.4  20°C  78.6% | 7.4  20°C  78.6% |
| HDX time course analyzed (min) | 0.16, 1, 10, 30, 60, 120, 180 | 0.16, 1, 10, 30, 60, 120, 180 |
| HDX control samples | Fully-labeled control | Fully-labeled control |
| Number of peptides analyzed after labeling | 29 | 38 |
| Final sequence coverage after labeling | 100% | 100% |
| Average peptide length | 15.3 | 17.1 |
| Redundancy | 4.00 | 4.84 |
| Replicates | 3 (Technical) | 3 (Technical) |
|  |  |  |
| Repeatability (pooled standard deviation) ^*^ | 0.052 Da | 0.046 Da |
|  |  |  |
| Significant difference between state ^$^ | Wald test, *p* < 0.01 | |

^*^ One unique charge state was used per peptide

^$^ MEMHDX (www. memhdx.c3bi.pasteur.fr)

**Table S2: Cross-correlation coefficients for PulG pili models before and after real-space refinement**

| **3D density/model** | **initial** | **refined** |
| --- | --- | --- |
| PulG_WT_ | 0.765 ± 0.017 | 0.781 ± 0.004 |
| PulG_CC_ | 0.737 ± 0.009 | 0.738 ± 0.005 |
| PulG_CC_* | 0.781 ± 0.014 | 0.785 ± 0.005 |

* in low-pass filtered density at 7.5Å
